# Identification of FABP5 as an immunometabolic marker in human hepatocellular carcinoma

**DOI:** 10.1136/jitc-2019-000501

**Published:** 2020-07-01

**Authors:** Fangming Liu, Weiren Liu, Shuang Zhou, Chunhui Yang, Mengxin Tian, Guangshuai Jia, Han Wang, Bijun Zhu, Mingxiang Feng, Yan Lu, Tiankui Qiao, Xinxin Wang, Wei Cao, Xiangdong Wang, Yinghong Shi, Duojiao Wu

**Affiliations:** 1 Institute of Clinical Science, Zhongshan Hospital, Fudan University, Shanghai, China; 2 Liver Surgery Department of Zhongshan Hospital, Fudan University, Shanghai, China; 3 Clinical Center for Molecular Diagnosis and Therapy, The Second Affiliated Hospital of Fujian Medical University, Quanzhou, China; 4 Gracell Biotechnologies Co., Ltd, Shanghai, China; 5 Affiliated Cancer Hospital & Institute, Guangzhou Medical University, Guangzhou, China; 6 Thoracic Surgery Department of Zhongshan Hospital, Fudan University, Shanghai, China; 7 Endocrinology and Metabolism Department of Zhongshan Hospital, Key Laboratory of Metabolism and Molecular Medicine, the Ministry of Education, Fudan University, Shanghai, China; 8 Jinshan Hospital Center for Tumor Dignosis & Therapy, Jinshan Hospital, Fudan University, Shanghai, China

**Keywords:** CD8-positive T-lymphocytes, biomarkers, tumor, tumor microenvironment

## Abstract

**Background:**

Regulating T-cell metabolism is crucial for their anticancer activity. Therefore, understanding the function and metabolism of human tumor-infiltrating T cells is of broad interest and clinical importance.

**Methods:**

CD3^+^CD45^+^ T cells were sorted from adjacent area or tumor core of human hepatocellular carcinoma (HCC), then the clusters and heterogeneity of T cells were further interrogated by single-cell transcriptomic profiling. 118 surgical samples from patients with HCC were histologically examined for infiltration of CD8^+^ T cells in tumor and adjacent tissue.

**Results:**

Single-cell transcriptomic profiling indicated that several exhausted T-cell (Tex) populations differentially coexisted in the tumor and adjacent tissue. CD137 identifies and enriches Tex with superior effector functions and proliferation capacity. Furthermore, enhanced fatty acid-binding protein 5 (FABP5) expression along with increased mitochondrial oxidative metabolism were evident in these CD137-enriched Tex. Inhibiting FABP5 expression and mitochondrial fatty acid oxidation impaired the anti-apoptosis and proliferation of CD137-enriched Tex. These observations have been verified by generating CD137 CART. Immunohistochemistry staining on the tissue microarray of 118 patients with HCC showed intra-tumoral FABP5 ^high^ CD8^+^ T-cell infiltration was linked to overall and recurrence-free survival.

**Conclusions:**

The tumor microenvironment can impose metabolic restrictions on T-cell function. CD137, a costimulatory molecule highly expressed on some Tex, uses exogenous fatty acids and oxidative metabolism to mediate antitumor immunity. The immunometabolic marker FABP5 should be investigated in larger, longitudinal studies to determine their potential as prognostic biomarkers for HCC.

## Background

The principle that naturally occurring T cells with antitumor potential exist in human cancer has rationalized the application of immunotherapy in oncology. The association between intratumoral T-cell accumulation and improved survival in cancer predicts the critical antitumor role for tumor-speciﬁc T-cell activity.^1 2^ Exhausted CD8 T (Tex) cells are a distinct cell lineage that develop during chronic infections and cancers. Tex cells are characterized by progressive loss of proliferation, effector functions, upregulated and sustained co-inhibitory receptor expression, metabolic dysregulation, poor memory recall and homeostatic self-renewal, and distinct transcriptional and epigenetic programs.^3 4^ Reinvigorating cell function through blocking inhibitory receptors highlights the therapeutic potential of targeting Tex.^3^ Thus, understanding Tex in oncology is very important for designing novel immunotherapies.^1 2^

Metabolic activity, which both regulates and is regulated by cellular signaling pathways and epigenetics, profoundly influences the trajectories of immune cell differentiation, fate, and the immune response to tumors.^5 6^ For example, naive and memory CD8^+^ T cells use fatty acid oxidation (FAO) as a primary energy source to sustain their survival, while activated effector CD8^+^ T cells prefer glucose uptake.^7^ Therefore, understanding how cell metabolism influences T-cell antitumor activity is critical for designing strategies for improving the immune response to tumors.

Hepatocellular carcinoma (HCC) is the fourth most common tumor worldwide.^8^ Although patients with early-stage HCC benefit from latest treatments, up to 70% of patients may have disease recurrence at 5 years.^8^ A precise evaluation of the immune status of HCC is required to clarify and predict postoperative recurrence. Some studies demonstrate that the interplay between tumor cells and immune cells in microenvironment is critical for the HCC evolution and for the likelihood of response to immunotherapeutics.^9^ In this study, we performed single-cell RNA sequencing (scRNA) to explore the naturally tumor-infiltrating T cells and their spontaneous immune responses and metabolic regulation in HCC. We identified distinct Tex clusters that existed in HCC. Among them, CD137 (4-1BB, TNFSFR9)–stimulated Tex specifically altered their metabolic activity to fight metabolic restrictions on T-cell function in tumor microenvironment. CD137 is a TNFR-family member with costimulatory function that was originally identiﬁed as an inducible molecule expressed on activated CD8^+^ and CD4^+^ T cells.^10^ To evaluate the observed metabolic activity of CD137^+^CD8^+^ T cell in HCC microenvironment, we developed CD28 or CD137 CART cells to test the different effects of costimulation signaling. In comparison with CD28 CART, CD137 CART cells have enhanced lipid uptake and intracellular transport by upregulating fatty acid-binding protein 5 (FABP5) expression and mitochondrial oxidative metabolism. Inhibiting FABP5 expression in CD137 CART cells impairs their exogenous fatty acid uptake and the potential of persisting in HCC. By analyzing the clinical pathology and prognosis of 118 patients with HCC, we found that the presence of CD8^+^FABP5^+^ T cells of tumors was strongly correlated with recurrence rate. This study emphasizes the clinical relevance of FABP5 as an immunometabolic marker in HCC.

## Methods

### Human specimens

A total of 138 patients who were pathologically diagnosed with HCC were enrolled in this study. Paired HCC tumor and adjacent normal liver tissues were obtained from each patient. Formalin-fixed paraffin-embedded HCC specimens of 118 patients with HCC were developed into tissue microarrays; fresh samples of 20 patients were collected for the subsequent T-cell isolation and scRNA or in vitro test. None of the patients was treated with chemotherapy or radiation prior to tumor resection. The adjacent normal tissues were at least 3 cm from the matched tumor tissue. Besides, we collected fresh tumor and adjacent normal tissues of three patients with non-small cell lung carcinoma for some experiments in this study. All patients in this study provided written informed consent for sample collection and data analyses.

### Tissue microarrays (TMAs)

Formalin-fixed paraffin-embedded human HCC specimens were randomly collected from 118 patients with HCC at Zhong Shan Hospital (Shanghai, People’s Republic of China) between 2006 and 2007. TMAs were constructed by Shanghai Biochip, as described previously.^11^ The histopathological diagnosis was determined according to the WHO criteria. Tumor differentiation was graded using the Edmondson grading system.^12^ Tumor staging was based on the sixth edition of the tumor-node-metastasis (TNM) classification of the International Union Against Cancer. The clinicopathologic characteristics of 118 patients with HCC are summarized in online supplementary table S1. Follow-up procedures and postsurgical patient surveillance were described previously.^11 13^ TMAs were constructed by Shanghai Biochip, as described previously.^11^ Overall survival (OS) was defined as the interval between the dates of surgery and death. Time to recurrence (TTR) was defined as the interval between the dates of surgery and the dates of any diagnosed recurrence (intrahepatic recurrence and extrahepatic metastasis). For surviving patients, the data were censored at the date of death or final follow-up.

10.1136/jitc-2019-000501.supp1 Supplementary data

### Multiplex quantitative immunofluorescence

The multiplex quantitative immunofluorescence staining for TMA slides was performed as previously described.^14–17^ Slides were fluorescently stained with Opal 7-Color Manual IHC Kit (NEL811001KT) according to the manufacturer’s description. Multispectral images of arrays were acquired using Vectra Polaris multispectral imaging system (PerkinElmer), and quantitative positivity of primary antibodies was analyzed using inForm Tissue Finder software (PerkinElmer).

### Cell isolation and single-cell sequencing

Paired fresh tissues of cancerous and adjacent non-cancerous liver tissue were obtained during surgical resection. Tissues were put into RPMI 1640 containing 10% fetal bovine serum (FBS) and cut up into a slurry with sterilized surgical scissors followed by gentle rocking for 30 to 45 min in 37°C RPMI 1640 medium containing 0.1% (m/v) collagenase IV. Suspension was then filtered on a 40 µM strainer and centrifuged at 1100 rpm for 10 min. Supernatant was discarded. After erythrocyte lysis, the precipitate was washed and prepared for flow cytometry. Single-cell suspension was resuspended with FACS buffer containing 0.5% CD3 (BioLegend, Cat. No. 300308, Clone HIT3a) and 0.5% CD45RO (BioLegend, Cat. No. 304210, Clone UCHL1) antibodies, then incubated at 4°C for 30 min. After washing, cells were resuspended for fluorescent cell sorting. CD3^+^CD45RO^+^ T cells were sorted by using BD FACS Aria II.

For scRNA, isolated cells were counted in duplicate with a hemocytometer, diluted to 700–1200 cells/µL requiring a minimum cell viability of 70%. Single cells were separated on a chromium controller (10x Genomics, Pleasanton, California) following the manufacturer’s recommendations and a previous study.^18^ Library construction was performed using Single Cell 3′ Reagent Kits V2 (10x Genomics), which produces Illumina-ready sequencing libraries. After quality control by fragment analysis (AATI), libraries were sequenced by Illumina sequencer. Sequencing data from Illumina sequencer were processed with Cell Ranger pipeline (V.2.1.1; 10x Genomics) using default settings. Cell were only included if the number of expressed genes was greater than 200 and mitochondrial gene expression ratio was less than 5%. Gene expression matrix was normalized using log scale. Results were used for subsequent clustering analysis and visualized as a Principal component analysis (PCA).

### Generation of CART

The vector of anti-mesothelin chimeric antigen receptor (CAR) is constructed for the engineering of T cells to target human mesothelin with a 3^rd^ generation self inactivating lentiviral vector plasmid. Expression of the CAR transgenes was controlled under the EF-1α promoter. In the study, CART cells expressing CD137 or CD28 signaling domains are generated by Gracell Biotechnologies. The fully human anti-mesothelin CARs composed of a human mesothelin-specific single-chain antibody variable fragment (P4 scFv) coupled to T-cell signaling domains were constructed and evaluated. All CARs contained the SS1 scFv against human mesothelin protein. The mesothelin CAR was previously described.^19^ The CD28z CAR consisted of the scFv linked in cis to the intracellular domains of CD28 and CD3z through the CD8a hinge and a CD28 transmembrane domain, as described previously.^20^ Similarly, the BBz CAR contained the scFv linked to the CD137 intracellular portion and the CD3z domain through a CD8a hinge and transmembrane domain.^20^ ^21^

### CART activation

Mesothelin-expressing K562 cells were generated by transducing with a mesothelin-encoding lentiviral vector and collected in a freezing tube and immersed in liquid nitrogen for 5 min followed by a water bath at 37°C for 5 min. This step was repeated three to five times, then Trypan blue stain and microscopy were performed to ensure there are no live cells. After that, the fragments of mesothelin-expressing K562 cells were mixed with CD137 CART at a ratio of 2:1. Activated or unactivated CART were incubated at 37°C at a concentration of 1.2×10^6^/mL in X-VIVO15 Medium (Lonza, 04-418Q) containing 5% FBS. Then cells were collected after 24 hours for FACS analysis and 48 hours for RNA extraction. In order to evaluate the effects of metabolic restriction on CART function, CART cells were treated with TOFA (Acetyl-CoA Carboxylase inhibitor, 20 µM), UK5099 (mitochondrial pyruvate carrier inhibitor), GW9662 (PPARg inhibitor, 10 µM) during activation. Cells were collected after 24 hours for FACS analysis.

### FACS analysis

Cells were stained with CD3 (BioLegend, clone: HIT3a), CD8 (BioLegend, clone: SK1), CD45RO (BioLegend, clone: UCHL1), T-bet (BioLegend, clone: 4B10), Eomes (R&D Systems, clone: 644730), NFATC1 (BioLegend, clone: 7A6), PD-1 (BioLegend, clone: EH12.2H7), TIM-3 (BioLegend, clone: F38-2E2), CTLA4 (BioLegend, clone: L3D10), LAG3 (BioLegend, clone: 11C3C65), Bcl-XL (Abcam, clone:7B2.5), FABP5 (R&D Systems, clone: 311215)，CPT1α (Proteintech, 15184-1-AP), CD137 (BioLegend, clone: 4B4-1), mito Tracker Red CMXROS (Invitrogen, M7512), TMRM (Invitrogen, M20036), LIVE/DEAD Viability/Cytotoxicity Kit (Invitrogen, L3224), CD27 (BioLegend, clone: M-T271), CD127 (eBioscience, clone: eBioRDR5), IFNγ (BD Pharmingen, clone: B27), TCF7 (BioLegend, clone: 7F11A10), IRF4 (BioLegend, clone: IRF4.3E4), Blimp1 (BD Pharmingen, clone: 6D3), BCL2 (Cell Signaling Technology, clone: 124), Ki67 (Abcam, clone: B126.1), PPARγ (Abcam, clone: EPR18516), Annexin V (BD Pharmingen, 556547), CFSE (BD Pharmingen, 565082), KLRG1 (BioLegend, clone: 2F1/KLRG1), as well as BODIPY 493/503 (Invitrogen, D3922) and BODIPY FL C16 (Invitrogen, D3821). For intracellular staining, cells were given PMA/ionomycin (BioLegend, 423303) re-stimulation and then intracellular staining was performed as previously described.^6^ FACS analysis was performed on a BD FACS Aria II flow cytometer and analyzed by FlowJo V.6 software.

### Lactate dehydrogenase (LDH) cytotoxicity detection assay

CD137 CART cells respectively treated with TOFA, UK5099, and GW9662 were co-cultured with K562-mesothelin cell line. K562 cells were incubated at 37°C at a concentration of 1.2×10^4^/100 µL in X-VIVO15 Medium containing 5% FBS in 96-well plates. CD137 CART were co-cultured with K562 at a ratio of 10:1. Cells were collected after 24 hours and cytotoxicity was evaluated through CyQUANT LDH Cytotoxicity Assay (Thermo Fisher, C20301) according to the instruction.

### Ketone detection

CD3^+^CD8^+^CD45RO^+^ T cells from ANT or TM were obtained through cell sorting. The obtained cells were incubated for 24 hours and the supernatant was collected for ketone detection. The process was performed by Ketone Body Assay kit (Sigma-Aldrich, MAK134-1) according to the manual.

**Metabolic gene-expression analysis by RT-PCR**

qRT-PCR was used to quantify expression levels of certain candidate genes. Total RNA from cells was used as a template to synthesize cDNA with a PrimeScript RT Master Mix (TAKARA, RR036A). qRT-PCR was performed in sextuplicate with TB Green Premix Ex Taq II (TAKARA, RR820A) on a Roche LightCycler 480 System as per the manufacturer’s instructions. mRNA levels of each candidate gene as quantified by the PCR system were normalized to the housekeeping gene GAPDH. Primer sequences of genes are provided in online supplementary table S2.

10.1136/jitc-2019-000501.supp2 Supplementary data

### Metabolism assays

For real-time analysis of extracellular acidification rates and oxygen consumption rates (OCRs), CART (1.5×10^5^ cells/well) were analyzed using an XF-96 Extracellular Flux Analyzer (Seahorse Bioscience) as described in detail.^6^ For mitochondrial fitness tests, OCR was measured sequentially at basal, and then following the addition of 1 µM oligomycin, 1.5 µM FCCP (fluoro-carbonyl cyanide phenylhydrazone), 200 µM etomoxir, and 100 nM rotenone+1 µM antimycin A.

### Statistics of clinical characteristics

For research on the relationship between multi-labeled immunofluorescence results of TMAs and clinical information, contingency table analysis and χ^2^ tests were used. We simultaneously stained CD8, FABP5, lactate dehydrogenase A (LDHA) antibodies, and DAPI for cell nucleus. We counted positive rates of CD8^+^FABP5^+^, CD8^+^LDHA^+^ in duplicate for each dot. The averages of positive rates were calculated for every patient. Then positive rates of CD8^+^FABP5^+^, CD8^+^LDHA^+^ of tumor or peritumor TMAs were divided into high expression and low expression group in terms of cut point of OS judged by X-tile 3.5.0. Other clinical indicators were also divided into two groups according to certain clinical standards, as shown in online supplementary table S1. The χ^2^ test was conducted with SPSS statistics V.17 software. A p value <0.05 was considered statistically significant. For research of survival or recurrence rates, OS and time to recurrence (TTR) curves were plotted according to Kaplan-Meier in GraphPad Prism V.5. Basis for grouping of TTR was the same as aforementioned OS statistics. Life tables were performed using SPSS statistics to evaluate 1-year, 3-year, and 5-year survival and recurrence rates. For univariate and multivariate analysis of HR, a Cox regression analysis was performed with SPSS statistics.

### Statistical analysis of in vitro tests

Statistical analysis was performed using Prism 5 software (GraphPad). Comparisons for two groups were calculated using paired or unpaired two-tailed Student’s t-tests. Also, one-way ANOVA was used for multiple comparison.

## Results

### Composition of T cells in HCC

**Figure 1:**
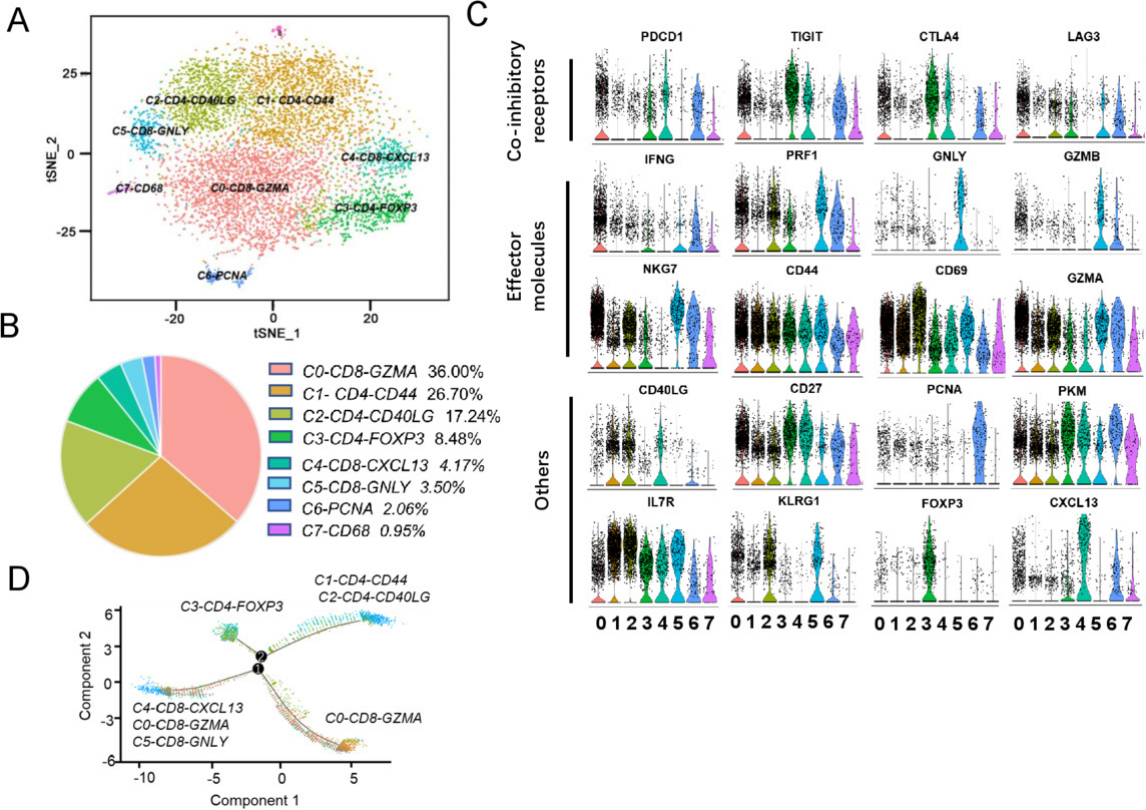
Clustering HCC-infiltrating T cells based on single-cell gene expression. (A) 2D visualization of single-cell clusters by t-SNE, showing the formation of 8 main clusters in different colors from integrated cell pool of HCC ANT and TM. (B) Pie chart showing the percentage of each cluster. (C) Violin plots showing the expression profile of multiple inhibitory receptors, cell markers, effector molecules in each cluster. (D) The branched trajectory of CD8^+^ T-cell state transition in a two-dimensional state-space inferred by Monocle (V.2). Each dot corresponds to one single cell, colored according to its cluster label. Arrows show the increasing directions of certain T-cell properties. ANT, adjacent non-cancerous tissue; HCC, hepatocellular carcinoma; TM, tumor core.

To generate a comprehensive view of the infiltrated T-cell ecosystem of HCC, we applied scRNA on 8047 sorted CD3^+^CD45RO^+^ cells from the HCC adjacent non-cancerous tissue (ANT) and tumor core (TM). *CD8A* and *IL-7R* were used as markers to classify CD8^+^ or CD4^+^ T cells. To reveal the intrinsic structure and potential functional subtypes of the overall T-cell populations, we performed unsupervised clustering of all cells using spectral clustering. For the integrated data, a total of eight stable clusters emerged, including three clusters for CD8^+^ cells and three clusters for CD4^+^ cells (figure 1A). We named each cluster and compared gene expression in figure 1A–1C. The percentage of each cluster is listed in figure 1B. We compared the expression of presentative genes coding co-inhibitory receptors, effector molecules, and other markers in figure 1C, online supplementary figure S1 and table S3.

10.1136/jitc-2019-000501.supp3 Supplementary data

10.1136/jitc-2019-000501.supp4 Supplementary data

Notably, C0-*CD8-GZMA* cluster is the largest cell population, accounting for 36.00% of the total cell. Cells of *C0* cluster expressed multiple known exhaustion marker genes such as *CTLA4*, *TIGIT*, *PDCD1*, and *LAG3*. Meanwhile, they overexpressed the effector genes *IFNG* and *GZMA* as well. The transcription signatures indicate that it is a cluster of partially exhausted effector CD8^+^ T cells. *C1-CD4-CD44* and *C2-CD4-CD40LG*, the two clusters, shared some mRNA signatures including *IL7R*, *CD44,* and *CD69* genes. When compared with *C1*, *C2* has higher expression level of killer cell lectin-like receptor G1 *KLRG1* (figure 1C). KLRG1 is a lymphocyte co-inhibitory, or immune checkpoint, receptor expressed predominantly on late-differentiated effector and effector memory CD8^+^ T and NK cells.^22^ Therefore, the *C2* is likely effector CD4^+^ T cell at a terminally differentiated state. Regulatory T cells (Tregs) were detected in the *C3-CD4-FOXP3* and accounted for 8.48% of total cells, highlighting the immunosuppressive nature of the tumor microenvironment of HCC. *C4-CD8-CXCL13* also expressed high levels of checkpoint genes *PDCD1*, *CTLA4*, *TIGIT*, and *CXCL13*. This leads us to conclude that both *C0* and *C4* clusters are exhausted and effector CD8^+^ T cells, and that the differentially expressed genes between the two clusters reflect heterogeneity and gradual development of CD8^+^ T-cell exhaustion. At the same time, the sixth cluster, *C5-CD8-GNLY*, highly expresses *GNLY*, *GZMB*, and *PRF1* which are typically natural killer T-cell markers. The seventh cluster, *C6-PCNA*, accounted for 2.06% of total cells, suggesting a small portion of proliferating T cells existed in HCC. Cells in the *C7-CD68* cluster expressed high levels of *CD68*, which is typically antigen expressed by tissue macrophages and cells of myeloid/mononuclear lineage. However, previous studies reported that CD68 antigen is constitutively expressed in NK cells and T cells and that its expression is strongly induced in activated CD4^+^ and CD8^+^ T lymphocytes.^23^ However, the character of *C7-CD68* needs further study. The heatmap of top genes in each cluster is shown in online supplementary figure S1.

Next, we analyzed CD4^+^ T and CD8^+^ T cells to determine the differentiation trajectory based on the pseudotime result (figure 1D). Although the major *C0-CD8-GZMA* cells were located in different directions with *C4-CD8-CXCL13*, *C5-CD8-GNLY* cells in the pseudotime trajectory plot, some *C0-CD8-GZMA* cells were mixed with them. Therefore, we inferred that *C0-CD8-GZMA* cells were more closely linked to intermediate populations between the effector populations (*C5-CD8-GNLY*) and the advanced exhausted population (*C4-CD8-CXCL13*). It remains to be seen if therapeutic strategies targeting these intermediate populations could prevent their transition to exhausted cells and instead promote them to effector cells. *C3-CD4-FOXP3*, *C1-CD4-CD44*, and *C2-CD4-CD40LG* were located at different branches, indicating functional divergence of these CD4^+^ T cells in HCC.

### Metabolic reprogramming of Tex is characterized by enhanced lipolysis and fatty acid oxidation

**Figure 2:**
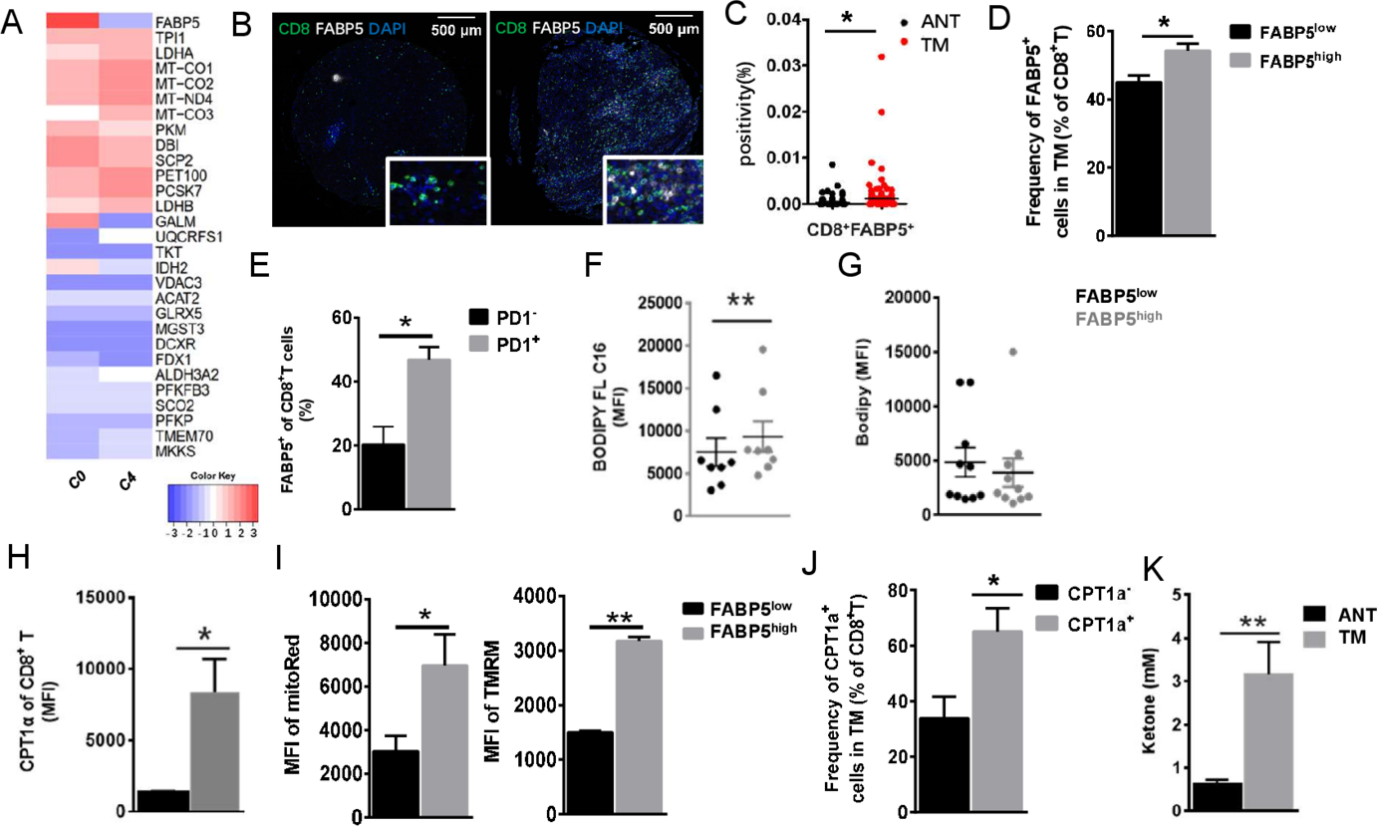
Increased expression of FABP5 in one Tex cluster. (A) Heatmap showing differentially expressed metabolic genes between two CD8^+^ Tex clusters. *C0* exhibited increased mRNA expression of *FABP5*. (B) Opal multicolor immunohistochemistry (IHC) staining with anti-CD8, FABP5 antibodies, and DAPI. Image on the left shows staining of ANT, while staining of TM is shown on the right. CD8, green; FABP5, white; DAPI, blue. (C) Positivity of CD8^+^FABP5^+^ cells in ANT and TM tissue array of 118 patients with HCC. Scatter diagram was obtained from positive rates of IHC staining. (D) The frequency of FABP5^+^ cells in CD8^+^ T cells of TM. (E) The frequency of FABP5^+^ CD8^+^ T cells in PD1^+^ or PD1^−^ cells of fresh HCC tumor. (F, G) MFI of lipid uptake and intracellular neutral lipid content (measured by BODIPY FL C16 and Bodipy), and (H) comparison of CPT1a protein level between FABP5^high^ and FABP5^low^ cells of CD3^+^CD8^+^CD45RO^+^ T cells. (I) Mitochondrial membrane potential measured by Mito Red and TMRM staining. (J) The frequency of CPT1a^+^CD8^+^ T cells in fresh HCC tumor and (K) the levels of ketone in supernatant of cultured CD3^+^CD8^+^CD45RO^+^ T cells isolated from ANT and TM. Data represent mean±SEM of reads from triplicate samples from one experiment and are representative of data from three experiments. p<0.05 was considered statistically significant. *p<0.05; **p<0.01. CPT1a, carnitine palmitoyltransferase IA; FABP5, fatty acid-binding protein 5; MFI, mean florescence intensity; TMRM, tetramethylrhodamine, methyl ester.

Although *C0* and *C4* clusters are both exhausted CD8^+^ T cells, we observed that they differentially expressed multiple important genes including *PRF1*, *IFNG*, *PCNA*, and so on. The difference reflects the heterogeneity of CD8^+^ T cells’ exhaustion in HCC. To further explore the detail mechanism, we compared the expression of metabolic genes in the two clusters (figure 2A). Although most genes are non-differentially expressed between them, we found that *FABP5* was specifically upregulated in cluster *C0* which has been identified as partially exhausted CD8^+^ T cells (figure 2A) and validated its expression on HCC-infiltrating CD8^+^ T cells by multiplexed immunohistochemistry (figure 2B–2C) and FACS analysis (figure 2D). Increased CD8^+^FABP^+^ T cells are infiltrated in TM instead of ANT (figure 2B–2C). Furthermore, the frequency of FABP5^high^ cells of CD3^+^CD45RO^+^CD8^+^ T cells was also higher in fresh TM (p<0.05, figure 2D).

Most of the FABP5^+^CD8^+^ T cells are PD1 positive (figure 2E). Given that FABP5 functions as a fatty acid-binding protein, we hypothesized that fatty acid metabolism might be different in CD3^+^CD8^+^CD45RO^+^FABP^high^ T cells of TM. Consistent with increased FABP5 levels, we determined that lipid uptake, measured by BODIPY FL C16 incorporation, was increased in CD3^+^CD8^+^CD45RO^+^FABP^high^ T cells of TM (figure 2F), although cellular neutral lipid content remained unchanged (figure 2G). Then carnitine palmitoyltransferase 1a (CPT1a), a rate-limiting enzyme of mitochondrial FAO which plays an important role in the utilization of fatty acids, was expressed at higher levels in CD3^+^CD8^+^CD45RO^+^FABP^high^ cells (figure 2H). Meanwhile, by using mitochondria probes (Mito Red and TMRM), we found that CD3^+^CD8^+^CD45RO^+^FABP^high^ cells have enhanced mitochondrial membrane potential (figure 2I). Also, the frequency of CPT1a^+^CD8^+^ T cells are accumulated in TM (p<0.05, figure 2J). The gating strategy of cells that were identified in measuring FABP5 and CPT1a is provided in online supplementary figure S2A. Consistent with the increased levels of CPT1a, a significant elevation of the ketone body 3-hydroxybutyrate, which is produced during FAO, was detected in culture supernatants from CD3^+^CD8^+^CD45RO^+^ cells of TM (figure 2K). Thus, the upregulated FABP5 in exhausted T cells is indicative of an increase in FAO, lipolysis, and utilization of exogenous fatty acids for β-oxidation. To verify that FABP5^high^CD8^+^ T cells are exhausted, we tested the expression of additional exhaustion markers (TIM3, CTLA4, and LAG3) in cells from tumor sample by flow cytometry. There is no significant difference of LAG3 protein level between FABP5^high^ and FABP5^low^ cells (online supplementary figure 2B). The protein level of TIM3 or CTLA4 is below the detection level (data not shown).

10.1136/jitc-2019-000501.supp5 Supplementary data

### Level of *FABP5* expression defines distinct exhausted CD8^+^ T cell infiltrated in HCC

**Figure 3:**
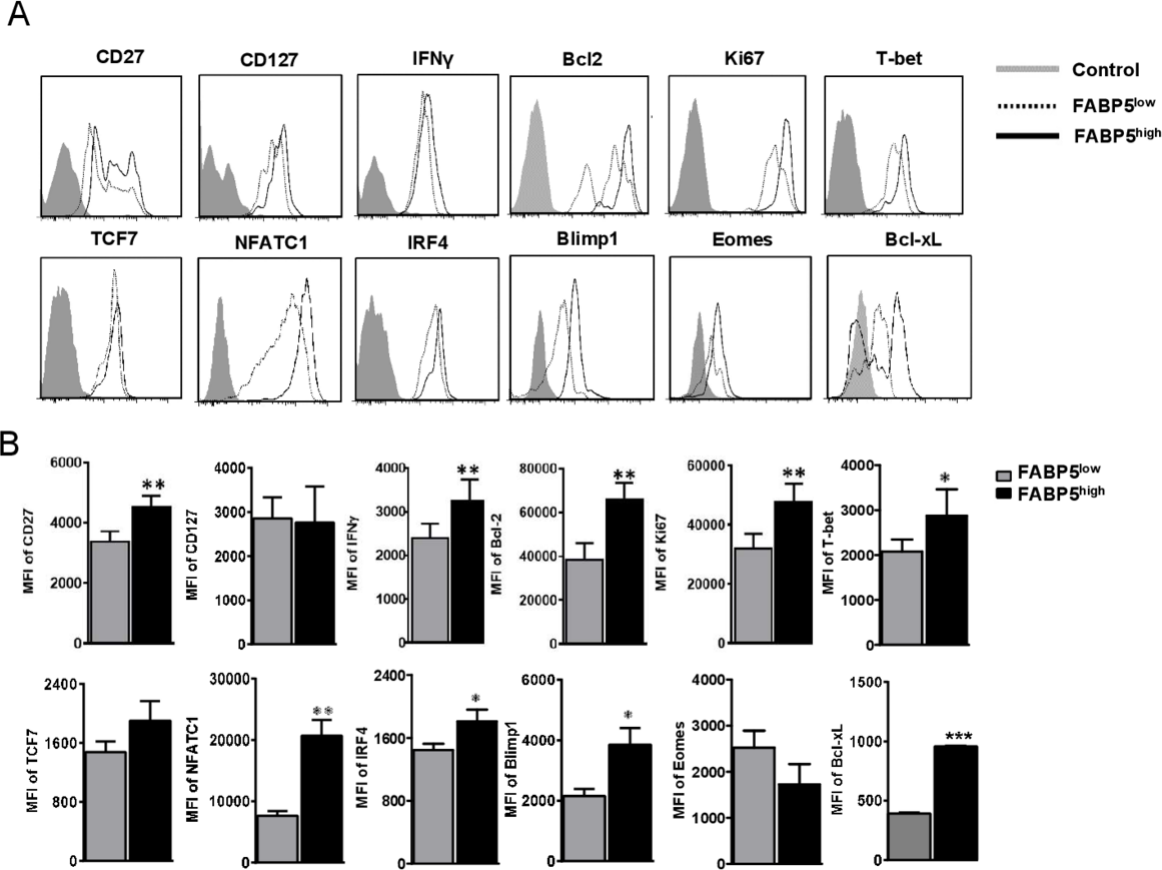
FABP5 expression defines two distinct Tex subpopulations of HCC. Flow cytometry was performed for patients with HCC to validate whether FABP5 expression defines different Tex of HCC. Exhausted CD8^+^ T cells of HCC tumor core were divided into two groups based on high/low FABP5 expression, then expression of CD27, CD127, IFNγ, Bcl2, Ki67, T-bet, TCF7, NFATC1, IRF4, Blimp1, Bcl-xL, and Eomes were compared. The representative flow cytometry figures and statistical data are shown in (A) and (B). Data are mean±SEM of reads from triplicate samples from one experiment, representative of 3–5. All differences shown are statistically significant (at least p<0.05 by Student’s t-test); p<0.05 was considered statistically significant. *p<0.05; **p<0.01; ***p<0.001.

We compared function genes of two CD8^+^ T-cell clusters within TM in figure 3. Notably, the FABP5^high^ CD8^+^ T cells increasingly expressed *IFNG* and *CD27*, which is required for generation and long-term maintenance of T-cell immunity^24 25^ (figure 3A–3B). Regarding transcription factors, the FABP5^high^CD8^+^ T cells expressed upregulated *T-bet*, *IRF4*, *NFATC1*, and *Blimp-1* that are typically associated with effector and exhausted CD8^+^ T cells, highlighting the distinct transcriptional fates of these two CD8^+^ T-cell subsets (figure 3A–3B). Besides, the protein level of anti-apoptosis and proliferation genes (*Bcl2*, *Bcl-xL*, *Ki67*) increased in FABP5^high^CD8^+^ T cells as well.

### Naturally upregulated FABP5 enriched in CD137-stimulated T cells

**Figure 4:**
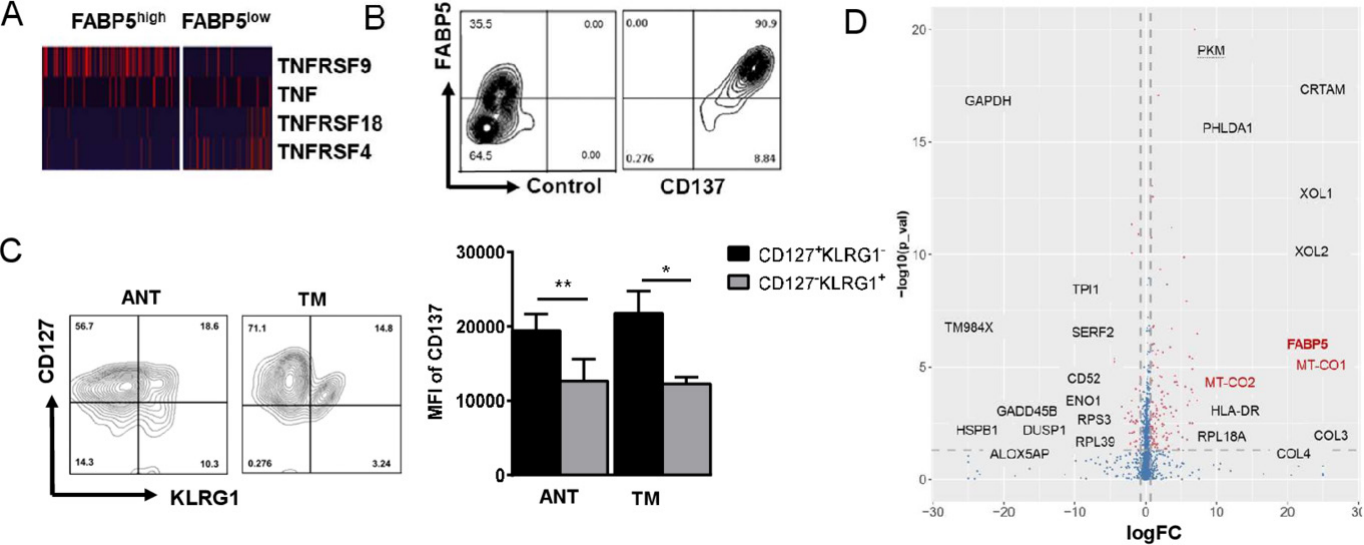
Enhanced FABP5 expression of CD137-enriched Tex. (A) Heatmap of scRNA showing differentially expressed genes of tumor necrosis factor (TNF) and TNF-receptor superfamily (CD8^+^FABP5^high^T cells vs CD8^+^FABP5^low^T cells). (B) Correlation of FABP5 and CD137 of CD8^+^ T cell in tumor core was analyzed through flow cytometry for patients with HCC. The left plot is isotype control of CD137 antibody. (C) Representative flow cytometry plots and MFI of CD137 in CD127^+^KLRG1^−^CD8^+^ T cells or CD127^−^KLRG1^+^CD8^+^ T cells of tumor core. (D) Volcano plot showing differentially expressed genes between the CD137^+^ and CD137^−^ populations. The plot was generated from scRNA data. Data are mean±SEM of reads from triplicate samples from one experiment, representative of 3–5. All differences shown are statistically significant; p<0.05 was considered statistically significant. *p<0.05; **p<0.01.

We found the mRNA level of TNFRSFs was distinct in the two CD8^+^ T-cell clusters (figure 4A). The total 85.33%±7.669% of FABP5^+^CD8^+^ T cells were TNFRSF9^+^ (CD137) cells, and we confirmed it on protein level (figure 4B). To verify whether the FABP5^high^CD8^+^ T cells and the features discussed previously were specific in HCC, we performed similar tests in dissected tumor or adjacent tissue of non-small cell lung cancer (NSCLC) in online supplementary figure S3. FABP5 expression presented in both adjacent tissue and tumor core of NSCLC. Similarly, FABP5 ^high^CD8^+^ T cells have higher amount of CD137, Bcl2, IFNγ, CPT1a, and increase lipid uptake (C16) in comparison with FABP5^low^CD8^+^ T cells in online supplementary figure S3. However, PD1 and Ki67 remain unchanged between two groups (data not shown). We observed similar changes between FABP5^high^ and FABP5^low^ cells from distinct locations. The enhanced expression of CD137, CPT1a, IFNγ, and PPARγ (except Bcl-2) were also found in FABP5^high^CD8^+^ T cells from peritumor tissue of NSCLC. Therefore, FABP5, as immunometabolic marker, has the potential to apply to multiple cancer types although we have no data regarding the clinical relevance of FABP5^+^ cells in NSCLC.

10.1136/jitc-2019-000501.supp6 Supplementary data

CD127^−^KLRG1^+^ and CD127^+^KLRG1^−^ are respectively used to define short-lived effector cells (SLECs) and memory precursor cells (MPECs).^26 27^ To evaluate the state of CD137^+^CD8^+^ T cells from HCC, CD127 and KLRG1 surface expression were detected by ﬂow cytometry. CD137 expression was significantly increased in CD127^+^KLRG1^−^ T cell (figure 4C).

Volcano plot showing differentially expressed genes between the CD137^+^ and CD137^−^ populations. Compared with the CD137^−^ population, CD137^+^CD8^+^ T cells highly expressed genes associated with metabolic functions such as *FABP5* and *Mitochondrially Encoded Cytochrome C Oxidase I* (*MT-CO1*), further supporting the finding that CD137^+^ cells were antigen-experienced and also induced metabolic reprogramming of CD8^+^ T cells (figure 4D).

### Effects of inhibiting FABP5 expression on T cells

**Figure 5:**
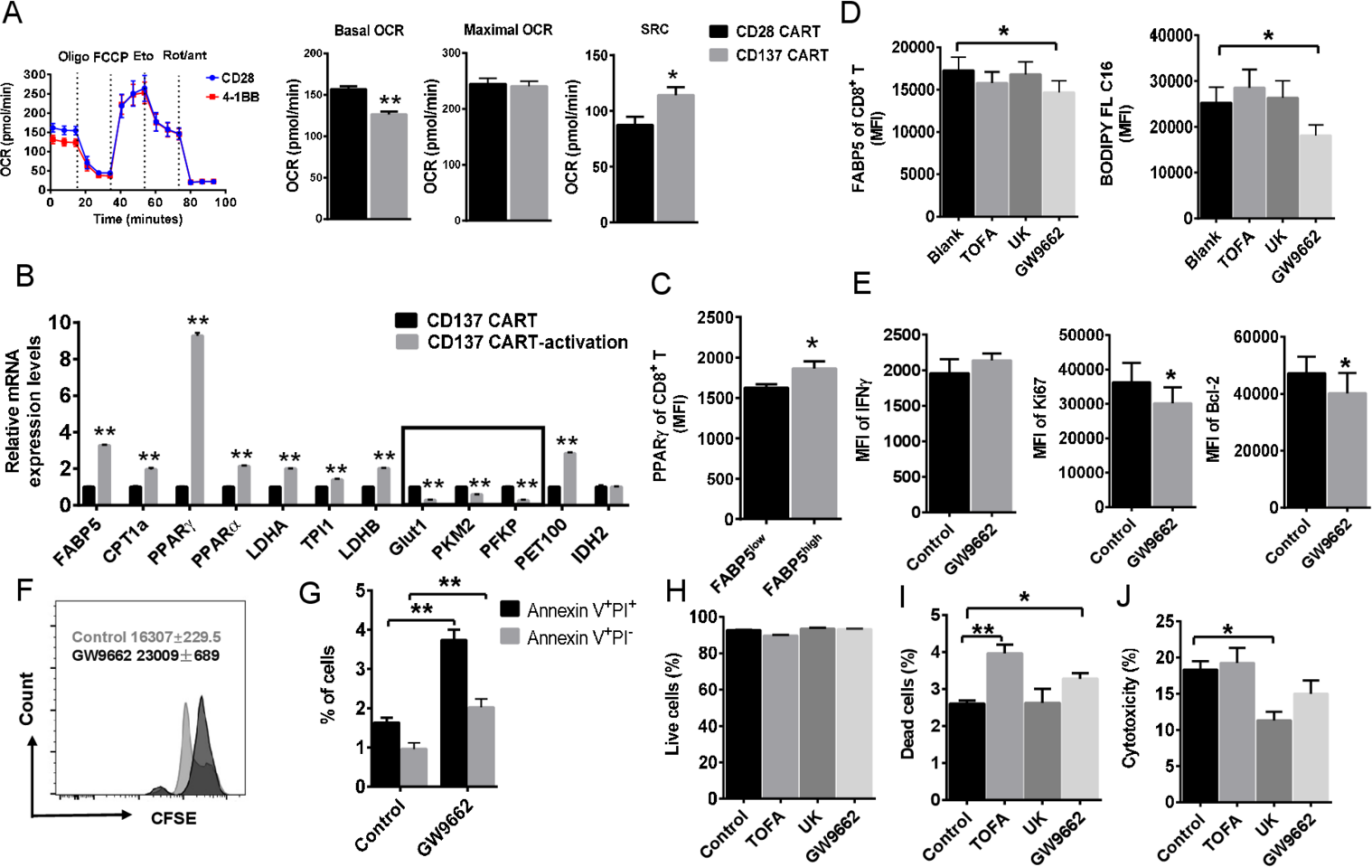
CD137 signaling instructs PPARγ-FABP5 expression. (A) Oxygen consumption rate (OCR) spectra of CD28 CART and CD137 CART using Seahorse XF96 analyzer. Basal OCR, maximal OCR, and spare respiratory capacity (SRC) were compared. (B) The expression of multiple metabolic genes in activated CD137 CART using real-time PCR. (C) The expression level of PPARγ of tumor-infiltrating CD8^+^ T cells was compared between FABP5^high^ and FABP5^low^ cells. (D) Cell suspension of HCC tumor tissue was treated with TOFA (10 µM), UK-5099 (50 µM), and PPARγ inhibitor GW9662 (10 µM) for 24 hours. Then expression of FABP5 and lipid uptake (measured by BODIPY FL C16) of CD8^+^ T cell was detected by cell flow cytometry. (E) After treatment of GW9662 for 24 hours, MFI of IFNγ, Ki67, and BCL2 in CD137 CART cells was detected by flow cytometry. (F, G) CFSE proliferation assays, Annexin V, and PI staining were also performed in CART cells treated with GW9662. (H, I) Survival of CD137 CART cells affected by metabolic intervention. We treated the activated CD137 CART cells with metabolic inhibitors for 24 hours and then performed live/dead staining. (J) The effects of energy deprivation on in vitro tumor-killing capability of CD137 CART cells. CD137 CART cells respectively treated with metabolic inhibitors and co-cultured with K562-mesothelin cell line at a ratio of 10:1. After 24 hours, cytotoxicity was evaluated through LDH cytotoxicity assay. Data represent mean±SEM of reads from triplicate samples from one experiment representative of 3. p<0.05 was considered statistically significant. *p<0.05; **p<0.01. PPARγ, peroxisome proliferator-activated receptor γ.

To conﬁrm that these metabolic alterations were due to the signaling domains of the receptor, similar experiments were performed with mesothelin-speciﬁc CART cells. We generated CD28 CART and CD137 CART. CD28 CART has higher basal OCR than CD137 CART cells (figure 5A). However, there was an increase in spare respiratory capacity (SRC) that was speciﬁc to the CD137 CART cells (figure 5A), following decoupling of the mitochondrial membrane using carbonyl cyanide-4-(trifluoromethoxy)phenylhydrazone (FCCP). Increased SRC likely supports T-cell function in a hostile tumor environment.^6 7^

To gain insight into the mechanism leading to the metabolic differences conferred by CD137 signaling domain, we measured the expression of candidate genes that are implicated in glycolytic and lipid metabolism (figure 5B). Primer sequences of genes implicated in glycolytic and lipid metabolism mentioned were provided in online supplementary table S2. Several genes involved in glycolysis are targets of hypoxia-inducible factor 1-alpha (HIF1α) including glucose transporter 1 (Glut1), phosphofructokinase (PFKP), and pyruvate kinase isozymes M2 (PKM2) were downregulated in stimulated-CD137 CART cells (figure 5B). Meanwhile, we found that genes associated with mitochondrial FAO were upregulated (figure 5B). In addition, mRNA levels of *FABP5*, which plays a critical role in long-chain fatty acid uptake, transport, and metabolism, were signiﬁcantly upregulated in CD137 CART cells. These ﬁndings suggest that CD137 programs T cells to use fatty acids as the predominant energy source, then supports T-cell proliferation, survival, and function in a nutrient-limited tumor environment. We noticed that peroxisome proliferator-activated receptor gamma (PPARγ) was extraordinarily upregulated in CD137 CART cells after specific antigen stimulation. Meanwhile, the mRNA expression level of PPARα was increased, which is consistent with enhanced expression of lipid oxidative genes. Then we confirmed that the expression level of PPARγ of tumor-infiltrating CD8^+^ T cells was higher in FABP5^high^ cells (figure 5C). PPARγ has been proven as a major regulator of lipid metabolism–related gene expression which includes FABPs. So we thought it was possible that PPARγ instructed the upregulated FABP5 expression of CD137-activated Tex. Then we use PPARg inhibitor GW9662 to test whether PPARg is responsible for FABP5 expression of CD137 signaling. In comparison with TOFA, de novo fatty acid synthesis inhibitor, or UK-5099, the inhibitor of the mitochondrial pyruvate carrier (MPC), GW9662 treatment specifically inhibited the protein amount of FABP5 and lipid uptake (figure 5D). Then we tested whether blocking PPARγ has effects on important biological functions of CD137 CART. The results showed that the decreased Ki67 and Bcl-2 expression presented in GW9662 treatment (figure 5E). However, there was no difference between ±GW9662-treated cells in IFNγ production of CD137^+^CD8^+^ T cells from HCC (figure 5E). Consistent with the Bcl-2 and Ki67 change, Annexin V-PI and CFSE proliferation assays support that the inhibition of FABP5 expression leads to impaired anti-apoptosis and proliferation (figure 5F–5G). To test the effects of metabolic deprivation on CART cell survival and cytotoxic function, we treated the activated CD137 CART cells with metabolic inhibitors TOFA, UK, and GW9662 for 24 hours, then measured the percentage of live/dead cells. TOFA and GW9662 treatments cause more dead cells although no significant statistical change was observed in live cells (figure 5H–5I). Therefore, fatty acid uptake and transport and fatty acid synthesis are both important for CD137 CART cell survival. In contrast, UK-5099, a specific MPC inhibitor, inhibited in vitro tumor killing of CART cells instead of cell survival as shown in figure 5H–5J. Thus, the effect of CD137 signaling–induced metabolic reprogramming contributes to long persistence of tumor-infiltrating lymphocytes (TILs) in tumor microenvironment rather than killing capacity.

### FABP5^high^ T cells of HCC are highly clinically relevant

**Figure 6:**
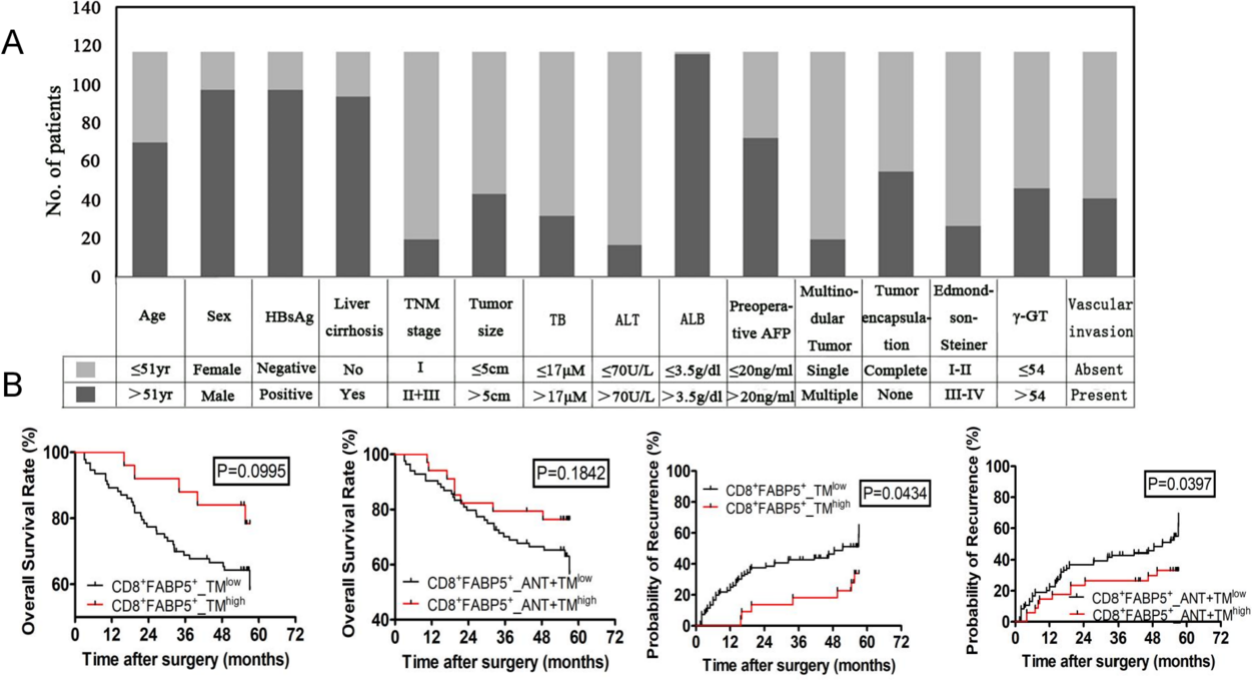
OS and TTR curves from ANT and TM tissue microarray. Based on cut points of OS status, data of OS and TTR status were divided into two groups (low vs high) judged by X-tile Kaplan-Meier analysis. p<0.05 was considered statistically significant. OS, overall survival; TTR, time to recurrence. CD8^+^FABP5^+^_TM^low^, low double positive expression of CD8 and FABP5 in TM; CD8^+^FABP5^+^_TM^high^, high double positive expression of CD8 and FABP5 in TM; CD8^+^FABP5^+^_ANT+TM^low^, low double positive expression of CD8 and FABP5 in both ANT and TM; CD8^+^FABP5^+^_ANT+TM^high^, high double positive expression of CD8 and FABP5 in both ANT and TM.

To evaluate clinical relevance of metabolic markers of the infiltrated T cells in HCC, we collected resected tumor and adjacent tissue from 118 patients and developed TMA. Clinical information of patients is summarized in figure 6A and online supplementary table S1. By the end of 5 years’ follow-up, 48.3% (57/118) of patients suffered a recurrence and 33.1% (39/118) died. The 1-year, 3-year, and 5-year OS rates were 81%, 70%, and 63% and the cumulative recurrence rates were 33%, 41%, and 56%, respectively. Tumor size, Edmondson-Steiner grade (III–IV vs I–II), γ-glutamyl transferase (γ-GT), BCLC stage system (B+C vs A), and vascular invasion were predictors of OS and TTR for univariate analysis. The individual clinicopathological features that presented significance in the univariate analysis were adopted as covariates in a multivariate Cox proportional-hazards model for further analysis. γ-GT was only one predictor for both OS and TTR in univariate/multivariate analysis (online supplementary table S1).

Except FABP5 discussed in this study, we added LDHA which is one of the top expressed metabolic genes among our scRNA data. LDHA has been reported that it is induced in activated T cells to support aerobic glycolysis and promotes IFNγ expression.^28^ We analyzed the correlation between the percentage of CD8^+^FABP5^+^ or CD8^+^LDHA^+^ T cells, overall survival rate, and cumulative recurrence rate by using Kaplan-Meier curve (figure 6B and Supplemental Figure S4). CD8^+^FABP5^high^T cells of patients with HCC seems to link with improved survival rate (figure 6B). However, p values are respectively 0.0995 and 0.1842 for TM and ANT+TM. Next, we found that CD8^+^FABP5^+^_TM^high^ predicts longer recurrence-free survival. Meanwhile, the cumulative recurrence rates of CD8^+^FABP5^+^_ANT+TM^high^ patients were also lower than that of CD8^+^FABP5^+^_ANT+TM^low^ (1 year: 34% vs 37%; 3 years: 30% vs 45%; 5 years: 36% vs 63%) (figure 6B). The data suggest that FABP5 has the potential of predicting clinical outcomes of HCC. Although LDHA plays an important role of affecting T-cell effector function, in our study, the protein level of LDHA in HCC tissue array has no correlation with patient survival and recurrence rate (online supplementary figure 4).

## Discussion

HCC is one of the leading causes of cancer-related death and is currently the main event leading to death in patients with cirrhosis.^29–31^ The metabolic syndrome with non-alcoholic liver disease may be an important cause of HCC in addition to viral hepatitis and alcohol-induced liver disease.^29 30^ The molecular pathogenesis is extremely complex and heterogeneous in HCC.^29 30^ Targeting T-cell metabolism in the tumor microenvironment is an important strategy for designing improved immunotherapy. In this study, the transcriptional profiles at single cell level provide new insight of studying metabolic heterogeneity of HCC-infiltrating T cells. We found in HCC that most TILs expressed multiple exhaustion markers (PDCD1, LAG3.). Among them, a cluster of CD137-stimulated Tex cells have stronger effector functions, survival, and anti-apoptosis characteristics. Of note, exhausted T cells are not inert. These cells retain suboptimal but crucial functions that limit ongoing pathogen replication or tumor progression. These properties were partially achieved by upregulating FABP5 expression and mediating lipid metabolic reprogramming that endow these cells with energetic advantages.

Persisting antigen stimulation,^32^ elevated and sustained expression of multiple inhibitory receptors,^33^ changes in metabolism,^34^ and a transcriptional program distinct from other T-cell differentiation states^27 32^ are signals that lead to progressive loss of T-cell effector function, resulting in poor control of pathogens or tumors. Meanwhile, although antibodies against PD-L1, TIM3, or LAG3 restore responses of HCC-derived T cells to tumor antigens,^35^ most patients do not experience durable clinical benefit.^36^ Currently, the important strategies to restore function in exhausted CD8^+^ T cells are under evaluation—some in combination with PD-1-targeted therapy.^31^ For example, a subset of stem cell–like PD-1^+^ CD8^+^ T cells responsible for the proliferative burst after PD-1 therapy has been recently described.^37^ A better understanding of Tex heterogeneity will have implications in the optimization of PD-1-directed immunotherapy in chronic infections and cancer.

To verify what we found in HCC infiltrating T cells, we generated CD28 CART and CD137 CART in vitro and compared their metabolic characteristics. They incorporate additional costimulatory cytoplasmic domains CD28 or 4-1BB (CD137) individually. These costimulation domains enhance proliferation and function of CART cells. In our study, the vector of anti-mesothelin chimeric antigen receptor is constructed for the engineering of T cells to target human mesothelin. Mesothelin is a differentiation antigen present on normal mesothelial cells and overexpressed in some human tumors. Compared with CD28 CART, CD137 CART has increased SRC which has been shown to be associated with long-term survival and stress tolerance of T memory cells.^7^ In response to antigen activation, lipid metabolism genes including FABP5 were significantly upregulated in CD137 CART. The upregulation of FABP5 was consistent with that of CD137^+^ Tex infiltrated in HCC, suggesting that CD137-costimulated T cells use exogenous free fatty acids and their oxidative metabolism to persist in tissue and to mediate protective immunity whether in tumor microenvironment or in vitro TCR ligation. CD137-costimulated T cells showed significantly overexpressed PPARγ and PPARα which were consisted with enhanced lipid uptake and lipid oxidation (figure 5B). Accordingly, inhibition of PPARγ reduced FABP5 expression and lipid uptake, while expression of cell proliferation and anti-apoptosis genes were also downregulated. It has been reported that Cpt1 and Bcl2 could form a complex to prevent apoptosis of CTLs.^38^ These observations suggest that the lipid metabolism reprogramming in exhausted CD8^+^ T cells impact the long-term fitness and survival of this CD8^+^ T-cell population. However, it should be noted that inhibiting FABP5 expression and lipid uptake does not have an impact on killing tumor cells, which suggests that the different biological features of Tex depend on distinct metabolic mechanisms.

On TCR activation, IL-7Rα (CD127) is initially downregulated on populations of activated effector cells and increased CD127 levels was shown to determine effector CD8^+^ T cells destined to become memory T cells.^25^ More recent evidence suggests that coordinate expression of CD127 and killer cell lectin-like receptor G1 (KLRG-1) distinguishes SLEC from those destined to develop into long-lived memory T cells. SLEC displays a KLRG-1^+^CD127^−^ phenotype, whereas MPECs exhibit a KLRG-1^−^CD127^+^ phenotype.^39^ In figure 4C, CD137^+^ Tex exhibit a KLRG-1^−^CD127^+^ phenotype which support their sustained presence in HCC, consistent with increased SRC. In addition, it is noteworthy that similar results were obtained from CD8^+^ tissue-resident memory T (TRM) cells. T-cell-specific deficiency of Fabp4 and Fabp5 (Fabp4/Fabp5) impairs exogenous free fatty acid uptake by CD8^+^ TRM cells and greatly reduces their long-term survival in vivo.^40^ Recent studies have highlighted the importance of fatty acid metabolism in maintenance of memory CD8^+^ T cells.^41 42^ FAO is therefore able to maintain NADPH and ATP levels and prevent ROS increase and cell death. This increased the ability of the cells to survive the surge in oxidative stress associated with a sudden increase in oxygen availability. The flexibility to switch between FA synthesis, lipid uptake, and degradation could be particularly important for T cells exposed to the limitation of nutrients and oxygen availability found in tumors.

## Conclusions

The current PD1/PDL1 inhibitor therapies assume that exhaustion might be reversed in Tex, or at least for a sufficiently large subset of this pool, suggesting that these cells are not terminally exhausted and can contribute to protective immunity if re-invigorated. Consequently, defining the complexity of Tex becomes extremely important as a predictor of checkpoint blockade treatment. The study highlights the clinical prognostic role of FABP5^high^ Tex, suggesting the potential application of metabolic biomarker in immune status assessment. Our study is a preliminary attempt to test the correlation between immunometabolic marker and clinical prognosis. The observations were found in both liver cancer and lung cancer, suggesting that it may be shared by different cancers.

10.1136/jitc-2019-000501.supp7 Supplementary data
