# Supplementary figures and images for: Identification of FABP5 as an immunometabolic marker in human hepatocellular carcinoma
Source: J Immunother Cancer. 2020 Jul 1;8(2):e000501. doi: 10.1136/jitc-2019-000501 (PMC7332195; doi:10.1136/jitc-2019-000501)

Supplemental Figure S1

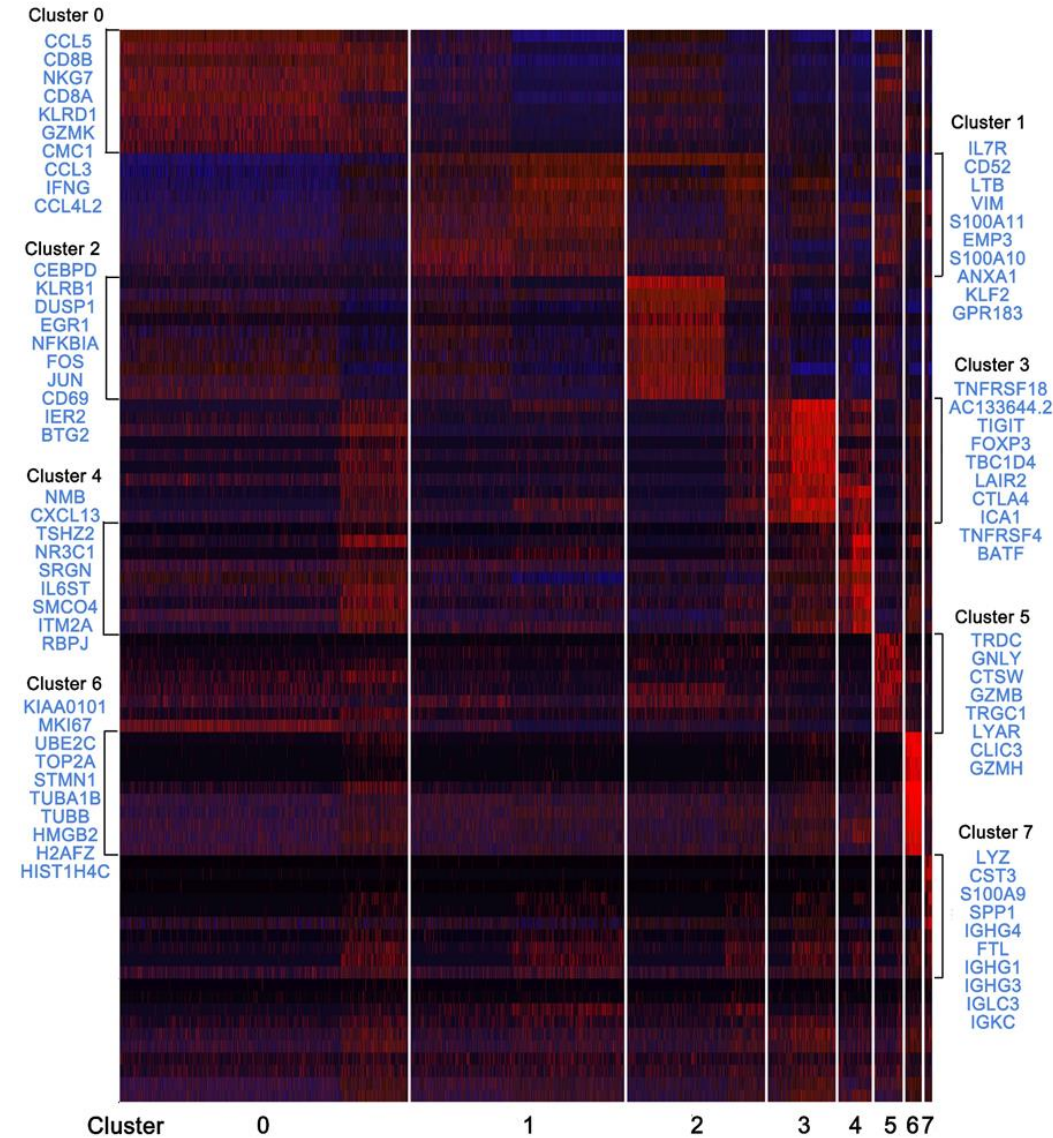

Supplement: Supplementary data [file jitc-2019-000501supp003.pdf]

Supplemental Figure S2

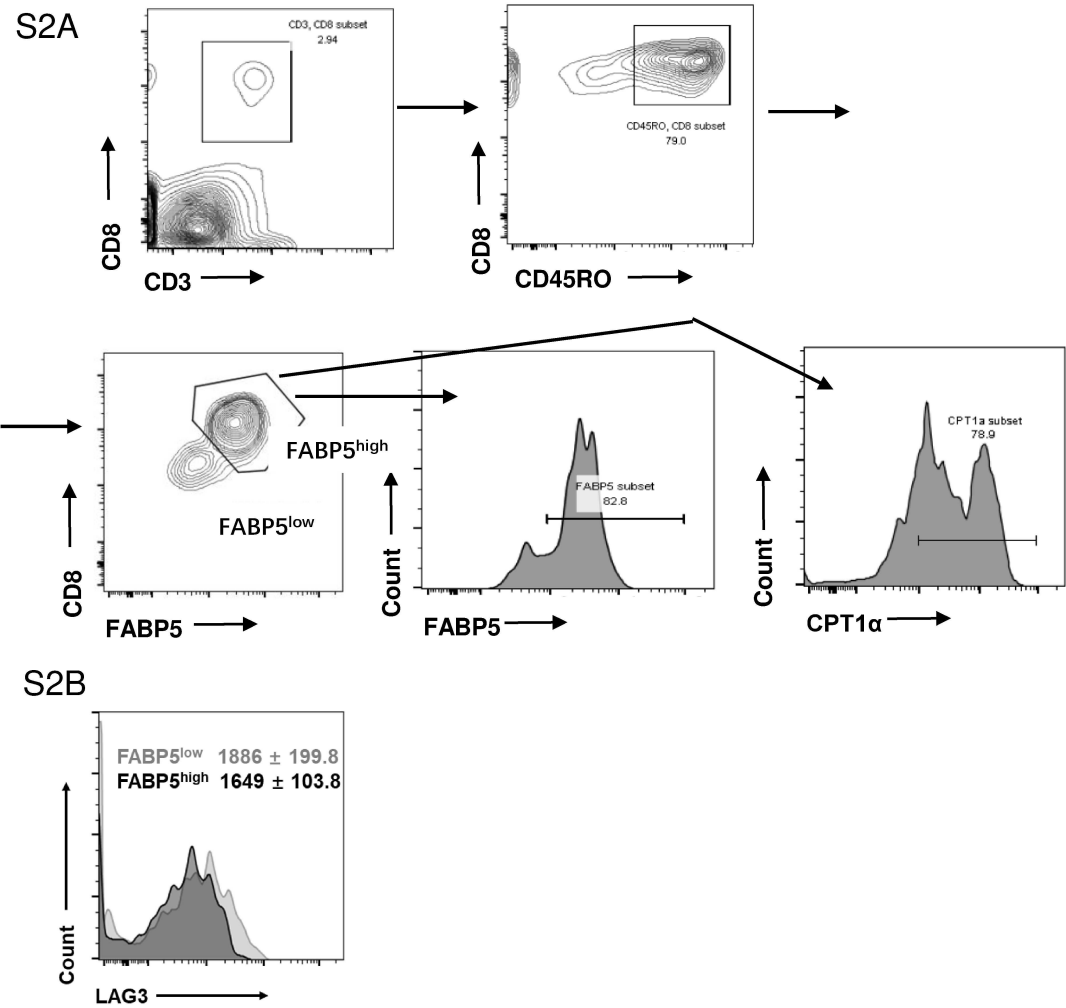

Supplement: Supplementary data [file jitc-2019-000501supp005.pdf]

Supplemental Figure S3

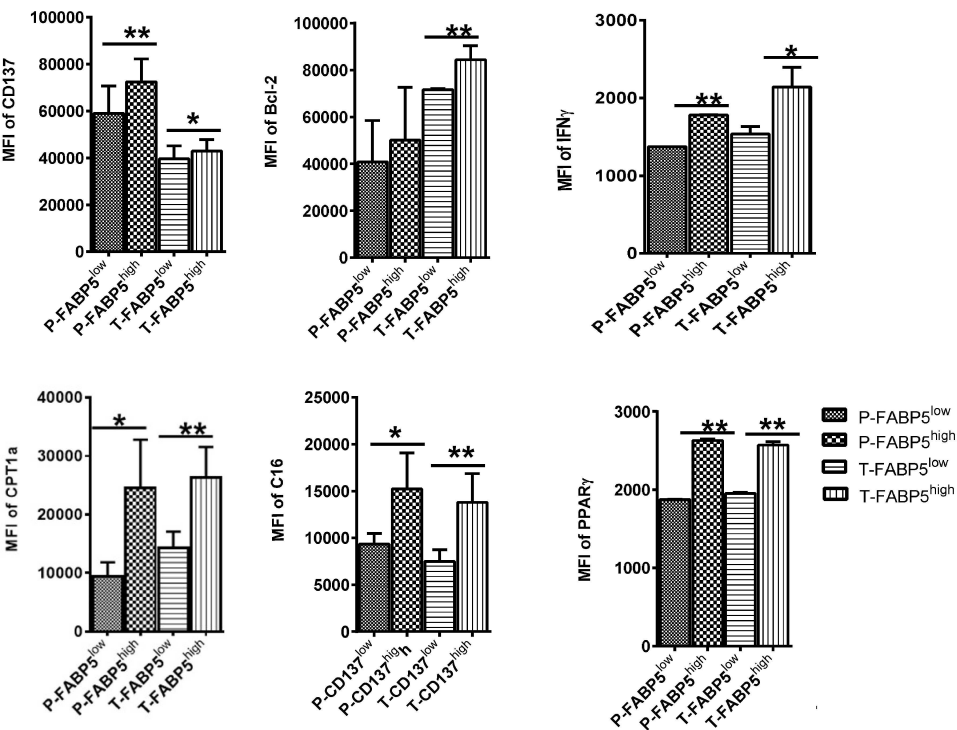

Supplement: Supplementary data [file jitc-2019-000501supp006.pdf]

Supplemental Figure S4

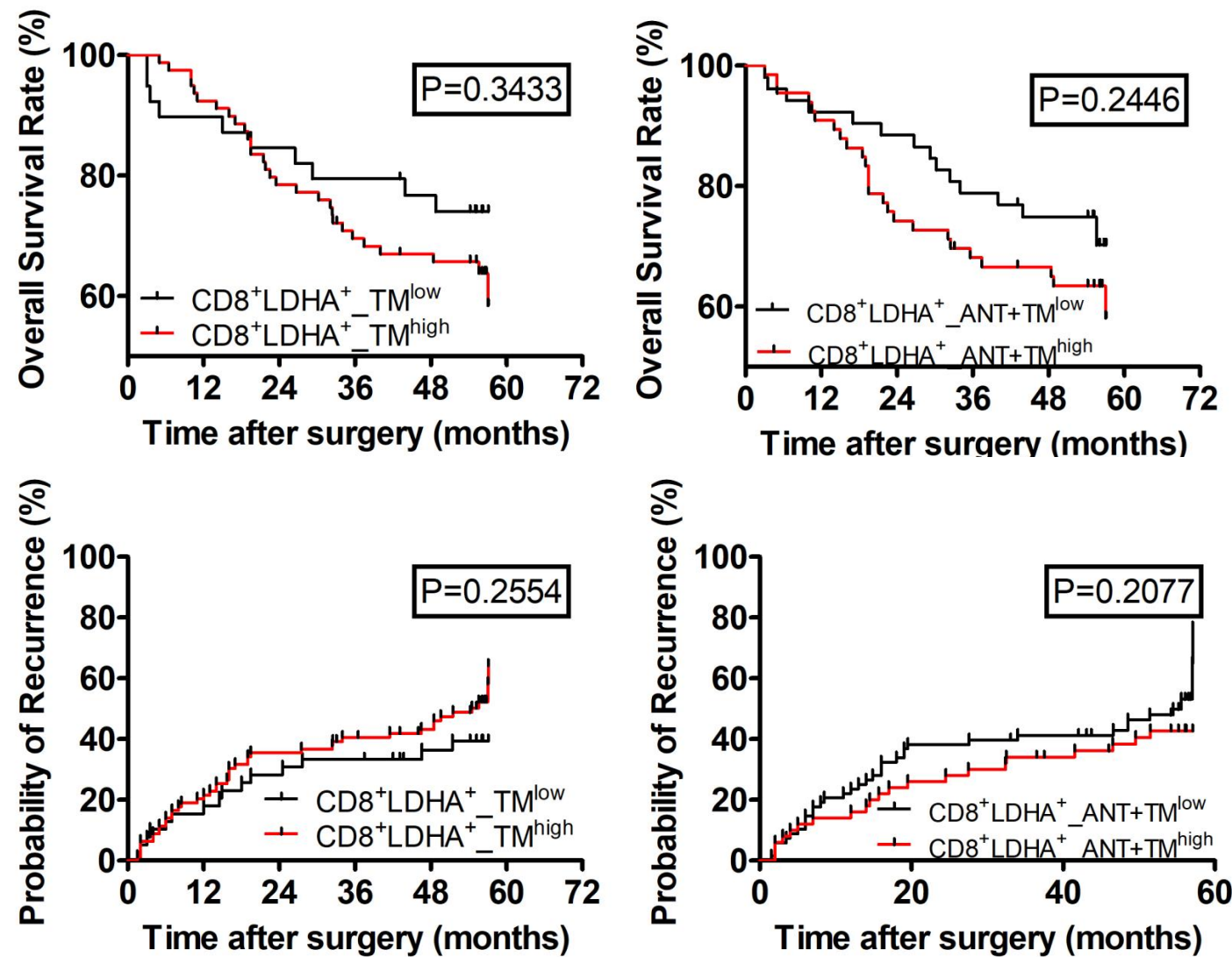

Supplement: Supplementary data [file jitc-2019-000501supp007.pdf]
