# Supplementary material for: Identification of FABP5 as an immunometabolic marker in human hepatocellular carcinoma
Source: J Immunother Cancer. 2020 Jul 1;8(2):e000501. doi: 10.1136/jitc-2019-000501 (PMC7332195; doi:10.1136/jitc-2019-000501)
Supplement: Supplementary data [file jitc-2019-000501supp004.pdf]

Table S3. Representative genes and functional description listed for each cluster of HCC.

Cluster No.	Tag	Representative genes	Functional description
**0**	C0-CD8-GZMA	GZMA,IFNG,TIGIT,NKG7,PDCD1	Cytotoxic and partially exhausted T cells
**1**	C1-CD4-CD44	IL7R,CD69, LTB,CD44	Activated T cells
**2**	C2-CD4-CD40LG	IL7R,CD69,CD40LG,KLRG1	T helper cells
**3**	C3-CD4-FOXP3	TIGIT,FOXP3,CTLA4,IL2RA	Treg
**4**	C4-CD8-CXCL13	CXCL13,PDCD1,CD27,CTLA4	Exhausted cells
**5**	C5-CD8-GNLY	GNLY,GZMA, NKG7,PRF1	Effector T cells
**6**	C6-PCNA	PCNA,MKI67,STMN1	Proliferating T cells
**7**	C7-CD68	CD68,C1QA,SERPINA1	Natural killer T cells

To generate a comprehensive view of the infiltrated T cells ecosystem of HCC, we applied single cell RNA sequencing on 8047 sorted $CD3^{+}CD45RO^{+}$ cells from the HCC adjacent non-cancerous tissue (ANT) and tumor core (TM). *CD8A* and *IL-7R* were used as markers to classify $CD8^{+}$ or $CD4^{+}$ T cells. To reveal the intrinsic structure and potential functional subtypes of the overall T cell populations, we performed unsupervised clustering of all T cells using spectral clustering. A total of 8 stable clusters emerged from the integrated data. We named each cluster and compared genes expression.
